# Results of a Randomized, Double-Blinded, Placebo-Controlled, Phase 2.5 Study of Saracatinib (AZD0530), in Patients with Recurrent Osteosarcoma Localized to the Lung

**DOI:** 10.1155/2020/7935475

**Published:** 2020-04-30

**Authors:** Kristin Baird, John Glod, Seth M. Steinberg, Denise Reinke, Joseph G. Pressey, Leo Mascarenhas, Noah Federman, Neyssa Marina, Sant Chawla, Joanne P. Lagmay, John Goldberg, Mohammed Milhem, David M. Loeb, James E. Butrynski, Brian Turpin, Arthur Staddon, Sheri L. Spunt, Robin L. Jones, Eve T. Rodler, Scott M. Schuetze, Scott H. Okuno, Lee Helman

**Affiliations:** ^1^Center for Cancer Research, NCI, NIH, Building 10 CRC, Room 1W-3750, MSC 110410 Center Drive, Bethesda, MD 20892-1104, USA; ^2^Center for Cancer Research, NCI, NIH, Building 10-CRC, Room 1-5750, Bethesda, MD 20892-1100, USA; ^3^Center for Cancer Research, NCI, NIH, 9609 Medical Center Drive, Room 2W334, MSC 9716, Bethesda, MD 20892, USA; ^4^SARC, 24 Frank Lloyd Wright Drive, Ann Arbor, MI 48106, USA; ^5^Cincinnati Children's Hospital Medical Center, 3333 Burnet Ave., MLC 7015, Cincinnati, OH 45229, USA; ^6^Children's Hospital Los Angeles, Keck School of Medicine, University of Southern California, 4650 Sunset Boulevard Mail Stop # 54, Los Angeles, CA 90027, USA; ^7^Mattel Children's Hospital, David Geffen School of Medicine, University of California, 10833 Le Conte Avenue, Los Angeles, CA 90095-6901, USA; ^8^Stanford University School of Medicine, Palo Alto, CA 94305, USA; ^9^Sarcoma Oncology Research Center, 2811 Wilshire Boulevard, Suite 411, Santa Monica, CA 90403, USA; ^10^University of Florida Health Shands Children's Hospital, 1600 SW Archer HD 204, Gainesville, FL 32610, USA; ^11^Dana-Farber Cancer Institute, 450 Brookline Ave, Boston, MA 02215, USA; ^12^University of Iowa Hospitals and Clinics, 200 Hawkins Drive C32 GH, Iowa City, IA 52242, USA; ^13^Children's Hospital at Montefiore, Albert Einstein College of Medicine, 3411 Wayne Ave., Room 910, Bronx, New York, NY 10467, USA; ^14^Willamette Valley Cancer Institute and Research Center, 520 Country Club Road, Eugene, OR 97401, USA; ^15^Cincinnati Children's Hospital Medical Center, 3333 Burnett Avenue, Cincinnati, OH 45229, USA; ^16^Abramson Cancer Center, University of Pennsylvania Health System, 230 W. Washington Square, Philadelphia, PA 19106, USA; ^17^Stanford University School of Medicine, 1000 Welch Road Suite 300, MC 5798, Palo Alto, CA 94304, USA; ^18^The Royal Marsden Hospital and Institute of Cancer Research, Fulham Road, London SW3 6JJ, UK; ^19^Comprehensive Cancer Center University of California, Davis, 2279 45th Street, Sacramento, CA 95717, USA; ^20^University of Michigan, 1500 East Medical Center Dr. Ann Arbor, Ann Arbor, MI 48109, USA; ^21^Mayo Clinic, 200 First Street SW, Rochester, MN 55905, USA; ^22^Children's Hospital Los Angeles, Keck School of Medicine, University of Southern California Children's Hospital Los Angeles, 4650 Sunset Blvd Mail Stop 57, Los Angeles, CA 90027, USA

## Abstract

**Purpose:**

Osteosarcoma is a rare cancer and a third of patients who have completed primary treatment will develop osteosarcoma recurrence. The Src pathway has been implicated in the metastatic behavior of osteosarcoma; about 95% of samples examined express Src or have evidence of downstream activation of this pathway. Saracatinib (AZD0530) is a potent and selective Src kinase inhibitor that was evaluated in adults in Phase 1 studies. The primary goal of this study was to determine if treatment with saracatinib could increase progression-free survival (PFS) for patients who have undergone complete resection of osteosarcoma lung metastases in a double-blinded, placebo-controlled trial. *Patients and Methods*. Subjects with recurrent osteosarcoma localized to lung and who had complete surgical removal of all lung nodules were randomized within six weeks after complete surgical resection. Saracatinib, or placebo, was administered at a dose of 175 mg orally, once daily, for up to thirteen 28-day cycles.

**Results:**

Thirty-seven subjects were included in the analyses; 18 subjects were randomized to receive saracatinib and 19 to receive placebo. Intent-to-treat analysis demonstrated a median PFS of 19.4 months in the saracatinib treatment group and 8.6 months in the placebo treatment group (*p*=0.47). Median OS was not reached in either arm.

**Conclusions:**

Although saracatinib was well tolerated in this patient population, there was no apparent impact of the drug in this double-blinded, placebo-controlled trial on OS, and Src inhibition alone may not be sufficient to suppress metastatic progression in osteosarcoma. There is a suggestion of potential clinical benefit as evidenced by longer PFS in patients randomized to saracatinib based on limited numbers of patients treated.

## 1. Introduction

### 1.1. Osteosarcoma

Osteosarcoma is the most common malignant bone tumor in the United States and Europe and occurs frequently in adolescents and young adults, as well as older adults (>70 years of age). Data from the National Cancer Institute's (NCI) Surveillance, Epidemiology, and End Results (SEER) report an estimated osteosarcoma incidence rate of 4.4 cases per 1 million in people aged 0 to 24 years [1]. The current 5-year survival rate is approximately 65% [2], and there has not been a substantial improvement in survival since the 1980s [3, 4]. Approximately one-third of patients who have completed primary therapy for localized osteosarcoma will develop recurrence and of those who develop recurrence, the five-year survival rate is approximately 25% [5, 6].

### 1.2. Src and Cancer

The proto-oncogene c-SRC (SRC), a member of the SRC family of protein tyrosine kinases, is a nonreceptor tyrosine kinase that mediates signal transduction affecting various cellular functions, including proliferation, differentiation, motility, adhesion, and survival [7–9]. Src can directly phosphorylate its substrates or act as a docking site for the binding of other signaling proteins that contain SH2 domains. Through this dual mechanism, Src directly and indirectly impacts multiple signaling pathways, including PI3K/AKT/mTOR, Ras/Raf/MEK/MAPK, and STAT3, all of which affect proliferation and survival of the cell. Src also regulates adhesions by targeting substrates including focal adhesion kinase (FAK) and paxillin [10, 11].

Increased Src activity was first described in sarcomas and is frequently implicated in cancer development. Examination of sarcoma tumor samples showed that 33% had enzyme activity levels that were 4- to 10-fold higher than that seen in normal tissue [12]. Similar findings were also found in mammary carcinomas [12]. Subsequently, increased activity or expression of Src was found in many common solid tumors, including the lung and several gastrointestinal tumors involving the esophagus, stomach, liver, pancreas, and colon [8]. In some cancers, Src activity correlates with poor prognosis.

### 1.3. Src and Osteosarcoma

Due to its aberrant expression, Src has been proposed to be important in signal transduction in human sarcomas, including osteosarcoma [13]. Total and phosphorylated Src have been found to be increased in several human sarcoma tissues including high-grade osteosarcoma and various sarcoma cell lines (osteosarcoma, Ewing's sarcoma, leiomyosarcoma, and rhabdomyosarcoma) [14]. Src activity has also been shown to be upregulated in anoikis-resistant human osteosarcoma cells, SAOS-2, when compared with their parental population [15]. In mouse models of osteosarcoma, depletion of Src phosphorylation in SaOS-2 cells leads to decreased tumor growth [16]. More recently published data from Urciuoli et al. demonstrated high levels of total and phosphorylated Src protein expression in osteosarcoma tissue samples and found that the subcellular location of expression may provide prognostic information [17].

### 1.4. Saracatinib and Osteosarcoma

Saracatinib (AZD0530) is a highly selective, orally bioavailable, dual-specific Src/Abl kinase inhibitor that has high potency against all Src family members tested [18]. In preclinical models and clinical studies, saracatinib modulates multiple key signaling pathways in cancer and inhibits osteoclast-mediated bone resorption [19–28]. Additionally, in vitro data show that Src plays an important role in the motility of osteosarcoma cells, a function that can be abrogated by the use of Src inhibitors [14]. More importantly, Src and other genes that are involved in the Src pathway are activated in 95% of patients with osteosarcoma [14, 17]. These data suggest that saracatinib may represent a promising therapy for the treatment of patients with recurrence of osteosarcoma.

## 2. Patients and Methods

### 2.1. Patients

From June 2009 to April 2014, subjects >15 years and <75 years of age with pulmonary recurrence of osteosarcoma who had complete surgical removal of all lung nodules or with suspected recurrence of osteosarcoma but had not yet had surgery were eligible for enrollment on “A Placebo-Controlled Study of Saracatinib (AZD0530) in Patients With Recurrent Osteosarcoma Localized to the Lung” (NCT00752206), which was a double-blinded, placebo-controlled trial. Presence of metastases was evaluated by CT chest and technetium bone scan. Those who enrolled prior to surgery were not randomized until inclusion and exclusion criteria were confirmed after surgery. Those not confirmed were considered screen failures and were not randomized. Randomization occurred within six weeks after complete surgical resection of all tumor nodules. Randomization was stratified by the number of recurrences (1^st^ vs. 2^nd^ vs. 3 or more) and lung metastases (1-2 vs. 3+). The Institutional Review Boards of all participating institutions approved the study, and all participants or their parent/guardian, as appropriate, provided written informed consent. The trial coordinating center was the SARC (Sarcoma Alliance for Research through Collaboration); all patients were registered electronically, and all adverse events were reported to SARC. Histological diagnosis of osteosarcoma (osteoblastic, chondroblastic, fibroblastic, or telangiectatic subtypes) in the metastases was required. Subjects must have previously received standard chemotherapy including doxorubicin, cisplatin, ifosfamide, and/or methotrexate. Subjects were excluded from enrollment if they had a recurrence at the primary site, metastatic disease in nonpulmonary sites, or extensive disruption of the pleura by tumor.

### 2.2. Study Aims and Treatment

The primary objective was to determine if the addition of saracatinib to pulmonary metastasectomy (S + PM) results in an increase in progression-free survival in this selected patient population. Additional secondary objectives were to determine if S + PM results in an increase in overall survival and time to treatment failure compared to placebo + PM.

**Figure 1:**
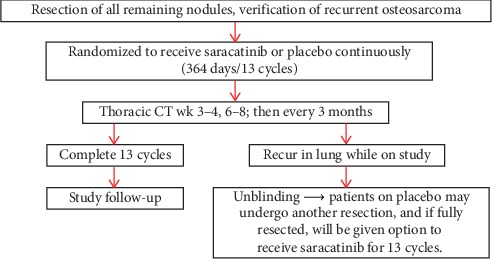
Treatment design schema. Schema is for subjects enrolled on NCT00752206, including the crossover design that was implemented in 2012.

Saracatinib, or placebo, was administered as a once-daily oral dose of 175 mg for 28 days per 28-day cycle for up to 13 cycles (364 days total) (Figure 1). Patients began cycle 1 after complete surgical resection of metastases. In February of 2012, a crossover design was added to improve enrollment after previous poor patient accrual. This study amendment allowed unblinding of patients who experienced isolated pulmonary recurrence of osteosarcoma that were considered to be amenable to complete surgical resection on study treatment. Those patients who were receiving placebo then had the option of receiving saracatinib following complete surgical resection. Saracatinib was administered similarly to those patients initially randomized to saracatinib.

### 2.3. Statistical Methods

The primary goal of this study was to determine whether the addition of saracatinib to pulmonary metastasectomy would result in an improvement in progression-free survival (PFS). The 2-year PFS probability was 33% when a second surgical complete remission was assumed [5]. The sample size was based on being able to detect a 60% relative improvement (from 33% to 53%) in PFS probability at two years. Assuming exponential survival curves, the hazard rate corresponding to this 2-year PFS probability for the control arm is 0.0462, which is defined as approximately a 0.0462 probability of failing each month when the 2-year PFS probability is 33%. If we assumed that the 2-year PFS probability was 53% for the saracatinib arm, the hazard rate is 0.0265, which then results in a hazard ratio of 1.75. Forty-four patients were required to be randomized in each arm of the study, for a total of 88 patients, over a 48-month accrual period to provide 80% power to detect a difference between the two resulting actuarial curves with a one-sided 0.10 alpha level log-rank test.

A secondary goal of this study was to determine whether the addition of saracatinib to surgery would result in an improvement in overall survival (OS). The 3-year OS probability is 45%, when a second surgical complete remission is achieved [5]. The sample size selected to evaluate PFS would be adequate to detect a 56% relative improvement (from 45% to 70%) in OS probability at three years. Assuming exponential survival curves, the hazard rate corresponding to this 3-year OS probability for the control arm is 0.0222, which is defined as approximately a 0.0222 probability of failing each month when the 3-year OS probability is 45%. If it was assumed that the 3-year OS probability may be 70% for the saracatinib arm, the hazard is 0.0099, which then results in a hazard ratio of 2.24. Evaluation of the 88 patients who were intended to be randomized over the same time frame would have provided 85% power to detect a difference between the two resulting actuarial curves with a one-sided 0.10 alpha level log-rank test.

## 3. Results

### 3.1. Efficacy

Forty-six subjects were enrolled during 2009–2014. Eight subjects were screening failures; therefore, 38 subjects were randomized to receive therapy. One randomized subject was subsequently taken off-study for pregnancy; therefore, 37 subjects were included in the analysis (Table 1). Most subjects were adolescent and young adults (AYA) with a median age of 22 years (range 15–55), and five were <18 years of age. The majority had osteoblastic subtype (*n* = 21), followed by chondroblastic (*n* = 9), telangiectatic (*n* = 4), and fibroblastic (*n* = 3). The median number of recurrences was 1 (mean = 1.75; range 1–3+), and the median number of lung nodules at enrollment was 1 (mean = 1.62; range 1–3+).

**Figure 2:**
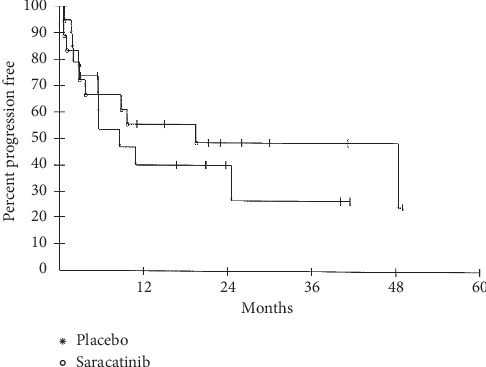
Progression-free survival. Intent-to-treat analysis demonstrated a median PFS of 19.4 months in the treatment group and 8.6 months in the control group.

**Figure 3:**
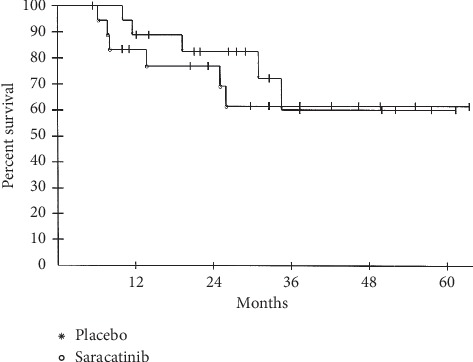
Overall survival. A median OS was not reached in either group. A 5 yr OS of 62% versus 60% in the treatment versus the control group was observed.

Eighteen subjects were randomized to receive saracatinib, and 19 were randomized to receive placebo. Nineteen subjects progressed while on study. Eighteen subjects completed therapy (saracatinib or placebo), 8 of those developed recurrence off therapy, while 10 remained disease-free at the time of analysis. Two subjects crossed over after progressing on placebo, and 2 withdrew from the study after progressing on placebo and declining crossover to saracatinib. With a data lock performed in October 2014, an intent-to-treat analysis demonstrated a median PFS of 19.4 months in the saracatinib treatment group and 8.6 months in the placebo treatment group, but no statistical difference (*p*=0.47 by log-rank test; Figure 2). Median OS was not reached in either group and the curves overlapped (*p*=0.61; Figure 3). The Data Safety Monitoring Board (DSMB) recommended study termination in 2014 for slow accrual and futility as no distinct impact of saracatinib on PFS or OS status postmetastasectomy was observed.

### 3.2. Safety

Overall, the regimen was well tolerated. A total of 358 adverse events occurred in 26 patients. These were mostly grade 1-2 events of minimal clinical significance, and 50% of those events were graded as possibly related to drug (laboratory abnormalities, gastrointestinal complaints, and pain). There were two grade 4 events that were unrelated to study drug and resolved completely. There were 20 grade 3 events reported: 11 were related to treatment and resolved completely. Of note, 4 subjects experienced grade 3 hypophosphatemia deemed to be related to saracatinib and requiring supplementation. There were no deaths on study.

## 4. Discussion

Although saracatinib was well tolerated in this patient population, there was no apparent impact of the drug in this double-blinded, placebo-controlled trial on OS. This mirrors several studies that also showed no effect of saracatinib as a single agent in other solid tumor types (non-small-cell lung cancer, colorectal cancer, thymic malignancies, and prostate) [29–32]. The observed toxicity profile in the present study was also similar to those observed in other published studies including hypophosphatemia requiring oral supplementation [30, 31].

Despite preclinical data that implicate the Src pathway in the development of pulmonary metastases in osteosarcoma, using Src tyrosine kinase inhibitors is likely insufficient to prevent recurrent pulmonary metastases following complete resection. In 2009, Hingorani et al. published results of their study that examined the effects of dasatinib, a dual Src-Abl kinase inhibitor, on in vitro proliferation, adhesion, and invasion of osteosarcoma cell lines and in preventing the development of spontaneous pulmonary metastases in an orthotopic murine osteosarcoma model. The authors found that although dasatinib inhibited Src and its downstream targets and inhibited the adhesion and migration of osteosarcoma cells in vitro, there was no impact on the development of pulmonary metastases in the murine model. They concluded that Src kinase activation might not be the primary pathway involved in the development of pulmonary metastases in osteosarcoma. However, they further concluded that Src inhibition combined with inhibition of one or more alternative pathways that are also implicated in the metastatic behavior of osteosarcoma (ezrin, insulin-like growth factor-I receptor pathway, and CXCR4) might be a rational approach for future clinical trials [33]. This is further supported by examples in the literature highlighting the complex pathways associated with Src signaling, summarized in a comprehensive review in 2015 by Liu et al. [34]. The authors conclude that the multifaceted role of Src in cancer metastasis and the relationship between Src and metastasis suppressors must be considered concurrently. They suggest that key metastasis suppressors such as N-myc downstream regulated gene 1 (NDRG1) play crucial roles in the effects of Src on the development of metastatic lesions and that additional therapeutic intervention targeting such suppressors may be a necessary component in antimetastatic therapy [34]. This is further evidenced by the results of a Phase 2 study of dasatinib in patients with previously treated, high-grade, advanced sarcoma (NCT00464620) where dasatinib failed to show activity as a single agent in the majority of sarcoma subtypes, including osteosarcoma [35].

One of the unique aspects of this trial, which was novel at the time of initiation, was the inclusion of subjects as young as 15 years of age. In 2009, when this study opened, it was among the earliest trials to include adolescents <18 years of age upfront and signaled the emerging acceptance of this approach from regulatory agencies, including Institutional Review Boards and the Food and Drug Administration (FDA). The success of this approach, in part due to this study, has led to several subsequent SARC trials enrolling subjects <18 years of age. This approach enables adolescents to have earlier access to drugs that may be beneficial and can potentially improve study accrual at sites that treat both adult and pediatric patients. Despite this, however, one of the major barriers in conducting this study was the ability to recruit subjects to this double-blind, placebo-controlled trial. Although 17 clinical centers opened this trial, enrollment was exceedingly slow, which prompted the change to the crossover study design. It is unclear whether the addition of the crossover design had an impact on this study, as enrollment did not appear to increase after the change. In fact, of the four subjects that progressed while on placebo, only 2 choose to crossover to saracatinib. Additionally, incorporating crossover designs into trial design can make certain endpoints, such as OS, difficult to interpret. While it is often required for New Drug Application approval from the FDA, it can be argued that introducing a double-blind, placebo-controlled study design prematurely may have adversely impacted study progression and drug development. Therefore, careful consideration of study designs in Phase 2 clinical trials is imperative to ensure adequate patient accrual and retention to ultimately yield optimal data collection.

## Figures and Tables

**Table 1:** Patient characteristics.

Male/female	19/18
Age (years)	Median 22 (range 15–55)
15–17	5
18–39	27
>40	5
Race	
Asian	4
Black	1
White	25
Unknown	7
Osteosarcoma subtype	
Chondroblastic	9
Fibroblastic	3
Osteoblastic	21
Telangiectatic	4
Number of recurrences	
1	19
2	8
3+	10
Number of lung nodules	
1	21
2	8
3+	8

## Data Availability

The data used to support the findings of this study are available from SARC upon request.
